# Re-evaluating the *MYH9* p.I1816V variant in a patient with atypical clinical presentation

**DOI:** 10.1007/s00467-025-07059-8

**Published:** 2025-11-17

**Authors:** Takao Konomoto, Fumito Wakamatsu, Hiromi Sakaguchi, Jun Kurogi, Etsuko Tanaka, Hiroshi Moritake

**Affiliations:** 1 Department of Pediatrics, Faculty of Medicine, University of Miyazaki, 5200 Kiyotake-Cho, Kihara Miyazaki City, Miyazaki Japan; 2 Department of Pediatrics, Miyazaki Prefectural Nichinan Hospital, 1-9-5 Kiyama, Nichinan City, Miyazaki Prefecture Japan

**Keywords:** MYH9-related disease, P.I1816V variant, Proteinuria, Next-generation sequencing, Genotype–phenotype discordance

## Abstract

MYH9-related disease (MYH9-RD) is an autosomal dominant disorder typically characterized by macrothrombocytopenia, leukocyte inclusion bodies, and variable non-hematologic manifestations such as hearing loss and nephropathy. We herein describe a 16-year-old boy presenting with persistent proteinuria and biopsy-proven membranous nephropathy with focal segmental sclerosis. Genetic testing identified a rare *MYH9* variant (p.I1816V), previously reported in association with Epstein syndrome. However, the patient had normal platelet counts, no leukocyte inclusions, and no abnormalities in non-muscle myosin heavy chain IIA (NMMHC-IIA) expression in neutrophils or podocytes. Although globally rare, the p.I1816V variant is more frequent in East Asian populations and is predicted to be benign by multiple in silico tools. This case illustrates the challenges of interpreting rare variants in the absence of supportive clinical findings and highlights the need for cautious evaluation in the era of next-generation sequencing.

**Supplementary information:**

The online version contains supplementary material available at 10.1007/s00467-025-07059-8.

## Introduction

MYH9-related disease (MYH9-RD) is a rare autosomal dominant disorder characterized by congenital macrothrombocytopenia, leukocyte inclusions, and variable non-hematologic features including hearing loss, cataracts, and nephropathy. While pathogenic *MYH9* variants typically present with hematologic findings, clinical expression is highly heterogeneous—even among individuals carrying the same variant.

Genetic testing is often performed in patients with unexplained glomerular disease, but interpretation can be challenging when a variant of uncertain significance (VUS) is identified and clinical features are atypical. The *MYH9* p.Ile1816Val (p.I1816V) variant is extremely rare and has only been reported in a family with Epstein syndrome and a patient with hearing loss [1, 2]. However, detailed phenotypic characterization is lacking, and its clinical significance therefore remains unclear.

Here, we describe a patient with proteinuria and kidney pathology showing membranous nephropathy (MN) with focal segmental glomerulosclerotic lesions, in whom panel-based genetic testing identified the p.I1816V variant. Importantly, the patient had no thrombocytopenia, leukocyte inclusions, or extra-renal features typically associated with MYH9-RD, and non-muscle myosin heavy chain IIA (NMMHC-IIA) staining was normal in both neutrophils and podocytes. This case underscores the limitations of genetic testing when supportive clinical findings are absent and highlights the need for cautious interpretation of rare variants.

## Case presentation

A 13-year-old boy was first found to have proteinuria during a routine school urinalysis. At 15 years of age, he was referred for evaluation of persistent proteinuria, obesity, and hypertension. His body mass index and blood pressure at the time were 31.5 kg/m^2^ (obesity: ≥ 25 kg/m^2^) and 137/78 mmHg (normal range for Japanese male senior high school students: 130/75 mmHg), respectively. Initial laboratory findings included a serum albumin level of 4.23 g/dL, a creatinine level of 0.61 mg/dL (normal range: 0.65–1.07 mg/dL), and an estimated glomerular filtration rate of 128 mL/min/1.73 m^2^. Urinalysis revealed 2 + proteinuria (urine protein/creatinine ratio: 1.04 g/gCr), with no occult blood. Serological tests for hepatitis B and C viruses, antinuclear antibody, and complement levels were all within normal limits. Evaluation for endocrine causes of obesity and hypertension revealed no abnormalities in thyroid or adrenal function. Kidney ultrasound was unremarkable. Proteinuria was initially attributed to obesity, and lifestyle modifications were recommended. However, because his weight and blood pressure remained stable, an angiotensin receptor blocker (ARB; valsartan 20 mg/day) was initiated at 16 years of age.

**Fig. 1:**
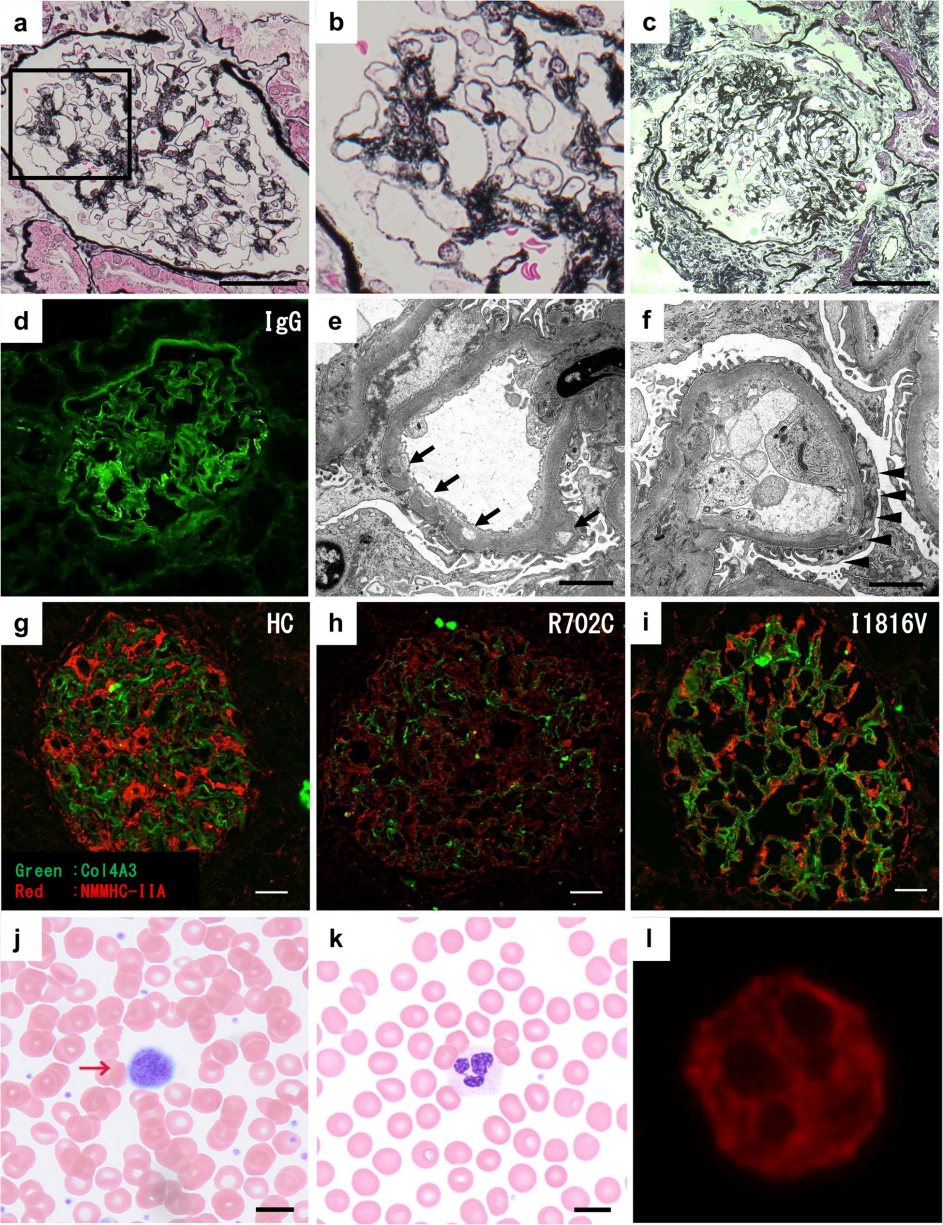
Peripheral blood smear and kidney biopsy findings. **a**–**c** Light microscopy of kidney tissue stained with periodic acid–methenamine silver, original magnification × 400, scale bar = 50 μm. **a** Segmental spikes and bubbling of the glomerular basement membrane are observed. **b** Enlarged view of the boxed area in **a**. **c** Focal segmental sclerotic lesions are observed in two glomeruli. **d** Immunofluorescence staining for IgG shows capillary wall deposition, whereas C3 deposition was absent. Original magnification × 400. **e**, **f** Electron microscopy of kidney tissue, original magnification × 3000, scale bar = 2 μm. **e** Thickening of the glomerular basement membrane with intramembranous lucent deposits (arrow). **f** Focal foot process effacement (arrowheads) is observed. **g**–**i** Immunofluorescence staining for NMMHC-IIA in kidney tissue, original magnification × 400, scale bar = 20 μm. Red: NMMHC-IIA; green: COL4A3 (Supplementary Fig. 2). Primary antibodies: rabbit polyclonal anti–NMMHC-IIA (PRB-440P, BioLegend; 1:100) and mouse monoclonal anti–COL4A3 (sc-52317, Santa Cruz; 1:50). Secondary antibodies: Alexa Fluor 555–conjugated anti-rabbit IgG (1:500) and Alexa Fluor 488–conjugated anti-mouse IgG (1:1000). **g** Control sample from a time-zero kidney transplant biopsy. **h** Markedly reduced NMMHC-IIA expression in podocytes from a patient with the severe p.R702C variant, compared with control. **i** No appreciable reduction in NMMHC-IIA expression in the current patient with the p.I1816V variant. **j**, **k** Peripheral blood smear stained with May–Giemsa, original magnification × 1000, scale bar = 10 μm. **j** Giant platelets (arrows) are observed, although only two were identified across all high-power fields (< 5%). **k** Döhle-like inclusion bodies are absent. **l** Immunofluorescence staining for NMMHC-IIA in neutrophils shows no abnormal cytoplasmic accumulation, original magnification × 1000

Because of persistent proteinuria, a kidney biopsy was performed at 18 years of age. Kidney pathology showed MN with focal segmental sclerotic lesions in a few glomeruli and mild hypertensive arteriosclerosis (Fig. 1a–c).

Immunofluorescence demonstrated IgG deposits along the glomerular basement membrane, and electron microscopy revealed localized thinning and thickening of the glomerular basement membrane with lucent intramembranous deposits, consistent with MN (Fig. 1d–f). Given the predominant IgG3 staining and negative M-type phospholipase A2 receptor staining, secondary MN was considered the most likely diagnosis, although staining for thrombospondin type-1 domain-containing 7 A and other antibodies was not performed. The presence of non-nephrotic-range proteinuria and focal foot process effacement suggested secondary rather than primary focal segmental glomerulosclerosis (FSGS).

Because no definitive secondary cause was identified—although obesity and hypertension could have contributed—panel-based genetic testing for hereditary FSGS was conducted. The analysis was performed at the Department of Pediatrics, Kobe University, using next-generation sequencing covering all exons and exon–intron boundaries of 153 genes associated with hereditary kidney diseases (Supplementary Table 1). This revealed a previously reported *MYH9* missense variant, NM_002473.6:c.5446A>G (p.Ile1816Val), originally described in a family with Epstein syndrome (Supplementary Fig. 1a).

Re-evaluation showed a normal platelet count (403 × 10^9^/L), but giant platelets were present with a prevalence of < 5% (Fig. 1j). Döhle-like inclusion bodies were not observed on May–Giemsa–stained peripheral blood smears (Fig. 1k). Immunofluorescence analysis of NMMHC-IIA was unremarkable in both neutrophils and glomerular podocytes (Fig. 1g–i, l). In addition, pure-tone audiometry showed no abnormalities, and no cataracts were detected. There was no family history of bleeding tendency, kidney disease, or hearing loss (Supplementary Fig. 1b). Family testing revealed no thrombocytopenia; however, the patient’s mother also exhibited giant platelets, again with a frequency of < 5%.

Based on these findings, MYH9-RD was considered unlikely. Given the presence of non-nephrotic proteinuria, absence of systemic autoimmune findings, and the possibility that pediatric MN can resolve spontaneously, we continued ARB therapy alone without initiating immunosuppressive treatment and lifestyle guidance was continued.

## Discussion

This case involved persistent proteinuria and a rare *MYH9* p.I1816V variant. However, typical hematologic features of MYH9-RD—macrothrombocytopenia, Döhle-like inclusions, and NMMHC-IIA abnormalities—were absent. Notably, giant platelets accounted for < 5% on smears, falling within the range observed in healthy controls and insufficient for a diagnosis of macrothrombocytopenia.

Macrothrombocytopenia is the hallmark of MYH9-RD and is typically the initial clinical manifestation that prompts diagnosis. Cases without thrombocytopenia are exceedingly rare, and even then, giant platelets and leukocyte inclusion bodies are usually present [3]. To our knowledge, no well-documented cases of genetically confirmed MYH9-RD have presented solely with kidney involvement. Our patient’s clinical features therefore do not convincingly support a diagnosis of MYH9-RD.

The p.I1816V variant is extremely rare globally (gnomAD frequency: 0.00000657) but shows higher frequencies in East Asians (0.0001929 overall and 0.0003441 in males in gnomAD; 0.000017 in ToMMo; 0.000318 in All of Us). These data argue against it being a highly penetrant pathogenic allele. In silico prediction tools also support a benign effect (Supplementary Fig. 1a). ClinVar currently classifies it as a VUS, and the few published reports describe inconsistent phenotypes—Epstein syndrome in one family and isolated hearing loss in another (although clinical details are limited). According to criteria established by the American College of Medical Genetics and Genomics and the Association for Molecular Pathology, the p.I1816V variant meets BS1 (allele frequency too high for a highly penetrant pathogenic variant) and BP4 (benign computational predictions) but lacks strong pathogenic evidence. Although ClinVar lists it as a VUS, our reassessment suggests that it is more consistent with a likely benign classification. Collectively, current evidence does not support pathogenicity; further clinical studies are needed.

In our case, immunofluorescence showed no abnormal NMMHC-IIA accumulation in neutrophils and no reduction in podocytes, unlike findings in the p.R702C variant. These results do not provide definitive evidence that the p.I1816V allele is pathogenic or sufficient to cause isolated nephropathy. Instead, this case illustrates the interpretive uncertainty that arises in genotype–phenotype discordance.

Kidney pathology in MYH9-RD remains poorly understood because of the limited number of biopsies, but reported findings are diverse and often include FSGS [4]. In this case, MN with FSGS was more likely unrelated to MYH9-RD and instead secondary to hypertension and/or obesity. The mechanisms of MYH9-related nephropathy remain unclear; for example, p.R702C causes a marked reduction of podocyte NMMHC-IIA, implicating direct podocyte injury [5]. Further case accumulation and functional validation are essential.

In conclusion, the increasing availability of next-generation sequencing may yield more cases in which genetic findings are difficult to interpret. Even when a variant is reported to be associated with a disease, it may not be the true cause in the absence of clinical validation or functional analysis. This case illustrates the limitations of genetic analysis and underscores the importance of cautious interpretation when genotype–phenotype correlations are unclear.

## **Summary**

### **What is new?**

- This case questions the pathogenicity of the *MYH9* p.I1816V variant and highlights the interpretive challenges of genotype–phenotype discordance, emphasizing the need for cautious evaluation of genetic findings.

## Supplementary information

Below is the link to the electronic supplementary material.

ESM 1 Supplementary Table 1(XLSX 13.7 KB)

ESM 2 Supplementary Figure 1(PDF 130 KB)

ESM 3 Supplementary Figure 2(PDF 414 KB)

## Data Availability

The data that support the findings of this case are available from the corresponding author.
