# Supplementary material for: Re-evaluating the MYH9 p.I1816V variant in a patient with atypical clinical presentation
Source: Pediatr Nephrol. 2025 Nov 17;41(4):993–7. doi: 10.1007/s00467-025-07059-8 (PMC12953253; doi:10.1007/s00467-025-07059-8)
Supplement: Supplementary file 2 — Supplementary Figure 1(PDF 130 KB) [file 467_2025_7059_MOESM2_ESM.pdf]

a: Gene analysis result

① NGS result

A heterozygous variant in *MYH9* :
NM_002473.6:c.5446A>G
(p.Ile1816Val),
based on the GRCh38/hg38

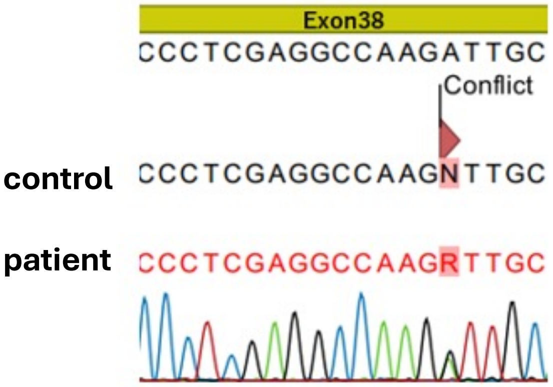

② In silico analysis

	SIFT	Polyphen2	CADD
c.5446 A>G	Tolerated	Benign	19.08

b: Family history and a three-generation pedigree chart

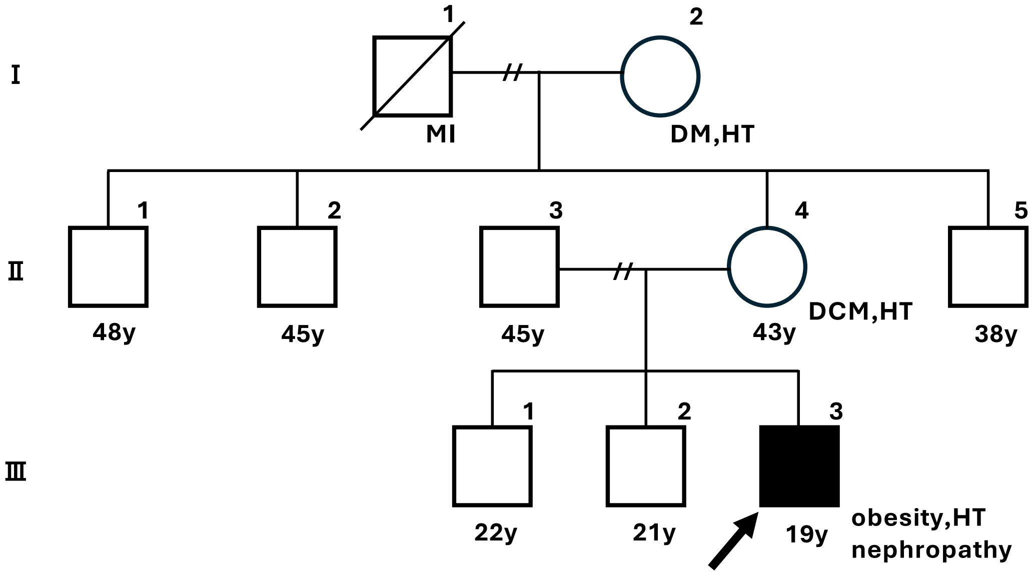

MI; myocardial infarction, DM; diabetes mellites, HT; hyper tension,
DCM; dilated cardiomyopathy
