# Supplementary material for: Re-evaluating the MYH9 p.I1816V variant in a patient with atypical clinical presentation
Source: Pediatr Nephrol. 2025 Nov 17;41(4):993–7. doi: 10.1007/s00467-025-07059-8 (PMC12953253; doi:10.1007/s00467-025-07059-8)
Supplement: Supplementary file 3 — Supplementary Figure 2(PDF 414 KB) [file 467_2025_7059_MOESM3_ESM.pdf]

Immunofluorescence staining for NMMHC-IIA in kidney tissue

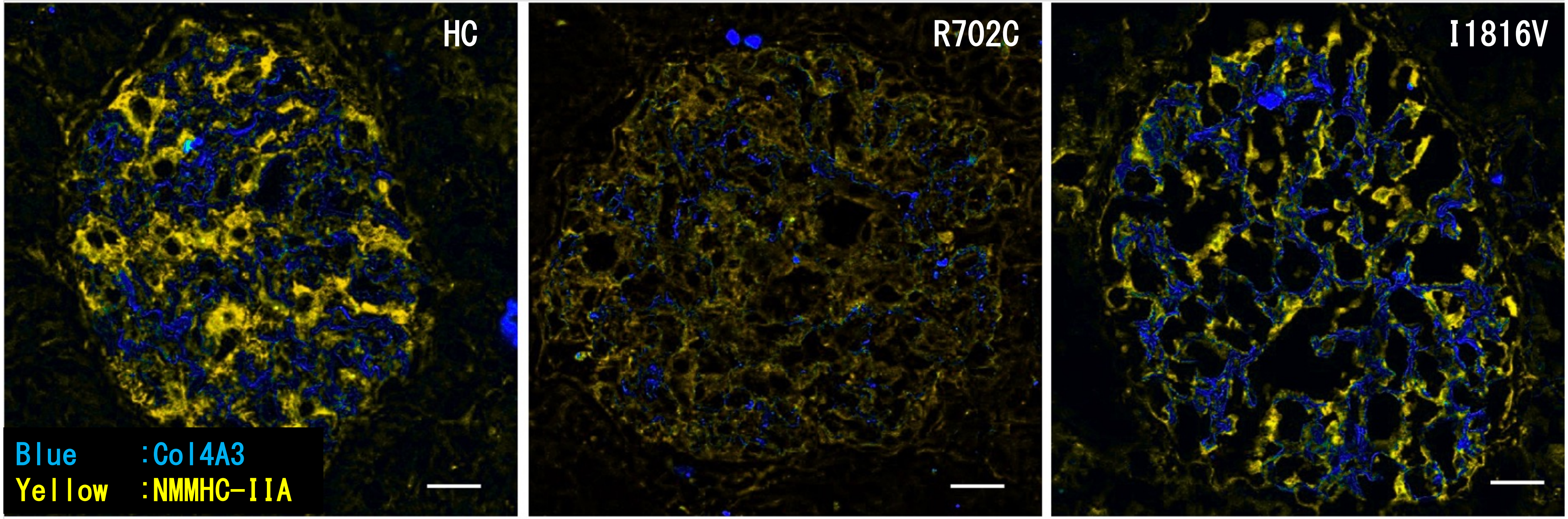

Original magnification ×400, scale bar = 20 μm
Yellow: NMMHC-IIA, Blue: COL4A3
